# A Continuous Description of Different Means with Application To Mixing Rules

**DOI:** 10.1021/acsomega.5c12377

**Published:** 2026-02-06

**Authors:** Uwe Hohm

**Affiliations:** Institut für Physikalische und Theoretische Chemie, 26527 TU Braunschweig, Gaußstr. 17, Braunschweig 38106, Germany

## Abstract

Consider two positive nonzero numbers *x*_1_ and *x*_2_. In many cases, the arithmetic (AM), geometric (GM), or harmonic mean (HM) is used as an appropriate mean *x*_12_ of *x*_1_ and *x*_2_: AM = (*x*_1_ + *x*_2_)/2, $\mathrm{GM}=\sqrt{x_{1}x_{2}}$ , and HM = GM^2^/AM. However, sometimes it is not clear from the outset which mean value should be selected. This results in a discrete problem. Instead of this, here we discuss three one-parameter functions, which are able to describe a continuous connection between the mean values HM, GM, and AM, respectively. Two of these functions are known as the Lehmer mean and Hölder mean, whereby especially the Hölder mean is suitable for generating a uniform one-parameter description of various mixing rules as used in quantum chemical force field calculations and thermophysical properties calculations.

## Graphical abstract

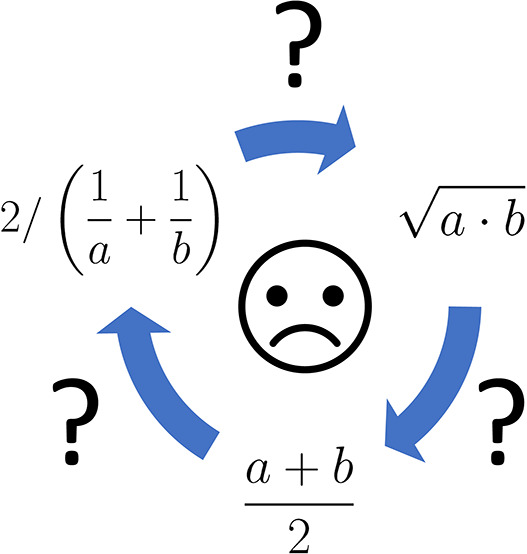

## Introduction

We consider two positive numbers *x*_1_ > 0 and *x*_2_ > 0. In many applications it is required to use some kind of mean value *x*_12_ of *x*_1_ and *x*_2_. In most cases the arithmetic mean AM = (*x*_1_ + *x*_2_)/2, the geometric mean GM = (*x*_1_ *x*_2_)^1/2^, or the harmonic mean HM = 2/(1/*x*_1_ + 1/*x*_2_) = GM^2^/AM are used. A predetermination of one of these three mean values is a discrete decision. Textbook examples from very different areas are the reduced mass μ = HM = 2/(1/*m*_1_ + 1/*m*_2_) of two vibrating masses *m*_1_ and *m*_2_, the formulation of the mean activity coefficient for a 1,1-electrolyte γ_±_ = GM = (γ_+_γ_–_)^1/2^, and the hard sphere collision diameter of two interacting particles *d*_12_ = AM = (*d*_1_ + *d*_2_)/2.^1^ Sometimes, however, it is not clear from the outset which mean is to be used.^2,3^ In this case it would be advantageous to vary continuously between the different mean values with a single continuous and differentiable function.

Here we consider the well-depth *ε*_12_ and collision-diameter σ_12_ or its equivalent measure *R*_*m*12_ of the intermolecular interaction potential *U*_12_ of two interacting particles 1 and 2. For isotropic systems these properties are well-defined. Despite the overwhelming success in the ab initio studies of the intermolecular interaction potential during the last two decades,^4^ a long-standing question still is how the interaction parameters *x*_12_ can be approximated from the neat properties *x*_1_ and *x*_2_, *x* = *ε*, σ, *R*_*m*_. To this end various mixing rules have been introduced. For example two of them are the well-known Lorentz–Berthelot mixing rules $\varepsilon_{12}=\sqrt{\varepsilon_{1}\varepsilon_{2}}$ and σ_12_ = (σ_1_ + σ_2_)/2. All of the mixing rules used have their advantages and drawbacks. However, the careful choice of the appropriate mixing rule is of central importance in the calculation of thermophysical properties as well as in the formulation of force fields applied to biomolecular modeling. Delhomelle and Millié^5^ claim that [···] *the choice of a set of combining rules has a significant effect on the thermodynamic properties* [···]. Dauber-Osguthorpe and Hagler^6^ state: *Combination rules: as important as parameters themselves*, whereas Horta et al.^7^ claim that among others one of the weakest component in classical force field representations is *[···] the application of ad hoc combination rules [···]*. Therefore, it would be desirable to have a parametrized function with a sound mathematical basis which allows for a continuous change between the different discrete mixing rules. In this contribution three different functions are considered: the Hölder mean, the Lehmer mean and a specially constructed mean value function. The first two means can be used to describe a large variety of different already existing mixing rules in terms of a continuous one parameter function. As an example we show that by using this new representation of mixing rules, experimentally determined cross second virial coefficients of binary noble gas mixtures can be reproduced particularly well. We will concentrate solely on this new continuous description of mixing rules. The combination of discrete already existing mixing rules with different potential energy functions was studied in depth by Kříž et al.^8^ and is not considered in this work.

## Theoretical

For a generalized description of the mean we consider three functions, two of which are already known in the literature. These two are the Hölder mean and the Lehmer mean, respectively, which, however, seem to be very rarely used in physicochemical applications. We restrict our analysis to two positive values with 0 < *x*_1_ < *x*_2_. These are the two numbers from which we want to calculate some sort of average *x*_1_ < *x*_12_ = *f*(*x*_1_, *x*_2_) < *x*_2_.

### The Hölder Mean

For any real number *p* ≠ 0 the Hölder mean (also known as power mean or generalized mean) *M*_*p*_(*x*_1_, *x*_2_) of two input data 0 < *x*_1_ < *x*_2_ is given by^9–12^

1 $$M_{p}\left(x_{1},x_{2}\right)={\left(\frac{1}{2}(x_{1}^{p}+x_{2}^{p})\right)}^{1/p}$$

The Hölder mean is directly related to the harmonic (HM) and geometric (GM) mean via

2 $$M_{-1}\left(x_{1},x_{2}\right)=\mathrm{HM}$$

3 $$M_{1}\left(x_{1},x_{2}\right)=\mathrm{AM}$$

In the limit *p* → 0 the geometric mean is obtained.

$$\underset{p\to 0}{\lim}{\left(\frac{x_{1}^{p}+x_{2}^{p}}{2}\right)}^{1/p}={\left(x_{1}x_{2}\right)}^{1/2}=M_{0}\left(x_{1},x_{2}\right)=\mathrm{GM}$$ 4

Combination of eqs 1 and 4 gives the continuous and differentiable function ([Disp-formula eq5]) which we will use here as a practical definition of the Hölder mean.

5 $$M_{p}\left(x_{1},x_{2}\right)=\{\begin{array}{lll} {\left(\frac{1}{2}(x_{1}^{p}+x_{2}^{p})\right)}^{1/p} & \mathrm{if} & p\neq 0\\ {\left(x_{1}x_{2}\right)}^{1/2} & \mathrm{if} & p=0 \end{array}$$

In the limits *p* → ±∞ we get

$$\underset{p\to -\infty }{\lim}M_{p}\left(x_{1},x_{2}\right)=x_{1}$$ 6

$$\underset{p\to +\infty }{\lim}M_{p}\left(x_{1},x_{2}\right)=x_{2}$$ 7

Moreover, the inequality (eq 8) holds for *p* < *q*

8 $$M_{p}\left(x_{1},x_{2}\right)<M_{q}\left(x_{1},x_{2}\right)$$

which immediately leads to the well-known inequality

9 HM<GM<AM

Some useful relations are

10 $$M_{p}\left(x_{1},x_{2}\right)M_{-p}\left(x_{1},x_{2}\right)=M_{0}^{2}\left(x_{1},x_{2}\right)$$

11 $$M_{p}\left(x_{1},x_{2}\right)/M_{p}\left(1/x_{1},1/x_{2}\right)=M_{0}^{2}\left(x_{1},x_{2}\right)$$

12 $$M_{p}\left(x_{1},x_{2}\right)/M_{p}\left(\sqrt{x_{1}/x_{2}},\sqrt{x_{2}/x_{1}}\right)=M_{0}\left(x_{1},x_{2}\right)$$

13 $$\begin{array}{lll} M_{p}\left(x_{1},x_{2}\right)M_{-p}\left(\sqrt{x_{1}/x_{2}},\sqrt{x_{2}/x_{1}}\right) & = & \\ M_{-p}\left(x_{1},x_{2}\right)M_{p}\left(\sqrt{x_{1}/x_{2}},\sqrt{x_{2}/x_{1}}\right) & = & M_{0}\left(x_{1},x_{2}\right) \end{array}$$

14 $$M_{p}\left(x_{1},1/x_{1}\right)M_{-p}\left(x_{1},1/x_{1}\right)=1$$

### The Lehmer Mean

A different mean value function is given by the Lehmer mean, p∈R :^9,10^

15 $${\mathrm{L̃}}_{p}\left(x_{1},x_{2}\right)=\frac{x_{1}^{p}+x_{2}^{p}}{x_{1}^{p-1}+x_{2}^{p-1}}$$

For *p* = 0, 1/2, and 1 we get the harmonic, geometric, and arithmetic mean, respectively. In order to make the index *p* congruent to the Hölder mean *M*_*p*_ we introduce the scaled Lehmer mean:

16 $$L_{p}\left(x_{1},x_{2}\right)=\frac{x_{1}^{p/2+1/2}+x_{2}^{p/2+1/2}}{x_{1}^{p/2-1/2}+x_{2}^{p/2-1/2}}$$

Lehmer mean and scaled Lehmer mean are related by

17 $${\mathrm{L̃}}_{p}=L_{2p-1}$$

According to eq 16 we obtain

18 $$L_{-1}\left(x_{1},x_{2}\right)=\mathrm{HM}$$

19 $$L_{0}\left(x_{1},x_{2}\right)=\mathrm{GM}$$

20 $$L_{1}\left(x_{1},x_{2}\right)=\mathrm{AM}$$

Therefore, for *p* = −1, 0, 1 the equality *M*_*p*_ = *L*_*p*_ holds. As for the Hölder mean the limits *p* → −∞ and *p* → ∞ give the minimum *x*_1_ and the maximum *x*_2_, respectively. Hölder and (scaled) Lehmer means are related by^9^

21 $${\mathrm{L̃}}_{p}\left(x_{1},x_{2}\right)=\frac{M_{p}{\left(x_{1},x_{2}\right)}^{p}}{M_{p-1}{\left(x_{1},x_{2}\right)}^{\left(p-1\right)}}$$

22 $$L_{p}\left(x_{1},x_{2}\right)=\frac{M_{(p+1)/2}{\left(x_{1},x_{2}\right)}^{(p+1)/2}}{M_{(p-1)/2}{\left(x_{1},x_{2}\right)}^{(p-1)/2}}$$

If all *M*_*p*_ are replaced by *L*_*p*_, the relationships given in eqs 10–14 are also valid for the scaled Lehmer mean.

### The Function *U_p_*

We present a third continuous mean-value function *U*_*p*_(*x*_1_, *x*_2_).

23 $$U_{p}\left(x_{1},x_{2}\right)={\left(x_{1}x_{2}\right)}^{\left(\frac{1-p}{2}\right)}{\left(\frac{x_{1}+x_{2}}{2}\right)}^{p}$$

Also in this case we find

24 $$U_{-1}\left(x_{1},x_{2}\right)=\mathrm{HM}$$

25 $$U_{0}\left(x_{1},x_{2}\right)=\mathrm{GM}$$

26 $$U_{1}\left(x_{1},x_{2}\right)=\mathrm{AM}$$

If all *M*_*p*_ are replaced by *U*_*p*_, eqs 10–14 are also valid for the function *U*_*p*_(*x*_1_, *x*_2_). It is very important to note, however, that in contrast to the Hölder and Lehmer means this function as a mean value does only make sense in the range −1 ≤ *p* ≤ + 1. If | *p* |> 1 *U*_*p*_ may lie outside the range (*x*_1_, *x*_2_) and so it does not have the meaning of a mean value. Nevertheless, it has been successfully applied as a mean in recent formulations of semiempirical electronic structure methods.^13^

### General Properties of the Hölder and (Scaled) Lehmer Mean and the Function *U_p_*

Some characteristic properties are summarized in Table 1. All three are continuous strictly increasing functions. Recall that the arithmetic mean is AM = (*x*_1_ + *x*_2_)/2, the geometric mean is GM = (*x*_1_ *x*_2_)^1/2^, and the harmonic mean HM = 2/(1/*x*_1_ + 1/*x*_2_) = GM^2^/AM. It was shown by Lehmer^10^ that for arbitrary *x*_1_ > 0 and *x*_2_ > 0 *M*_*p*_(*x*_1_, *x*_2_) and *L*_*p*_(*x*_1_, *x*_2_) do only coincide at *p* = −1, 0, + 1. The functions *M*_*p*_, *L*_*p*_, and *U*_*p*_ are exemplified in Figure 1.

**Figure 1:**
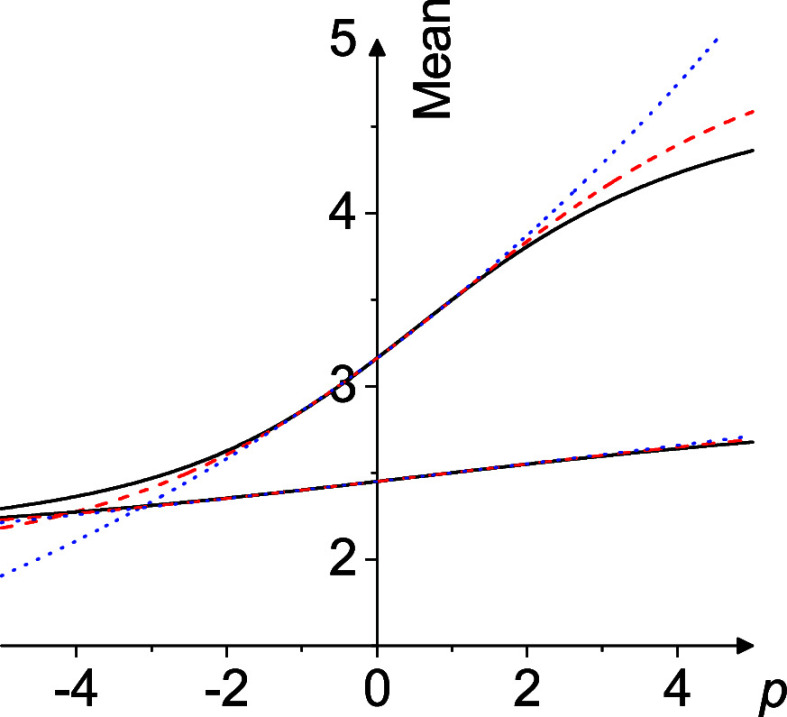
Hölder mean *M_p_* (*x*_1_, *x*_2_) (full black line), scaled Lehmer mean *L_p_* (*x*_1_, *x*_2_) (dashed red line), and *U_p_* (*x*_1_, *x*_2_) (dotted blue line) for *x*_1_ = 2 and *x*_2_ = 5 (upper triple) and *x*_1_ = 2 and *x*_2_ = 3 (lower triple) as a function of the parameter *p*. For *p* = −1, 0, + 1 the three functions always coincide exactly for arbitrary *x*_1_ and *x*_2_: *M_p_* (*x*_1_, *x*_2_) = *L_p_* (*x*_1_, *x*_2_) = *U_p_* (*x*_1_, *x*_2_).

**Table 1:** Characteristic Properties of *M_p_* (*x*_1_, *x*_2_) (eq 5), *L_p_* (*x*_1_, *x*_2_) (eq 16), and *U_p_* (*x*_1_, *x*_2_) (eq 23) for 0 < *x*_1_ < *x*_2_

	*M*_*p*_(*x*_1_, *x*_2_)	*L*_*p*_(*x*_1_, *x*_2_)	*U*_*p*_(*x*_1_, *x*_2_)
*p* → -∞	*x*_1_	*x*_1_	0
*p* → + ∞	*x*_2_	*x*_2_	∞
*p* = −1	HM	HM	HM
*p* = 0	GM	GM	GM
*p* = +1	AM	AM	AM
*p* < *q*	*M*_*p*_ < *M*_*q*_	*L*_*p*_ < *L*_*q*_	*U*_*p*_ < *U*_*q*_

By using the definitions in eqs 5, 16, and 23 we now have three continuous functions, which by changing the parameter *p* can sweep between the means HM, GM, and AM, respectively. As they are differentiable all three are particularly easy to use in optimization routines. Additionally, the Hölder mean and (scaled) Lehmer mean allow for | *p* | > 1. In this case, it is possible to calculate other mean values such as the quadratic (*M*_2_) or cubic mean (*M*_3_) in a consistent manner.

For application purposes it is important to note that in the vicinity of *p* = 0 the scaled Lehmer mean *L*_*p*_ and the function *U*_*p*_ are numerically much more stable than the Hölder mean. This is shown in Figure 2 where *M*_*p*_(32.8, 231.0), *L*_*p*_(32.8, 231.0), and *U*_*p*_(32.8, 231.0) are compared in the range −1.5 × 10^–10^ ≤ *p* ≤ 1.5 × 10^–10^. The numbers chosen in this example correspond to the potential well depths of neon and xenon, see Table 2. *L*_*p*_ and *U*_*p*_ show the expected smooth behavior. In contrast, *M*_*p*_ shows strong fluctuations with the used accuracy of 8 digits. This indicates that when using the Hölder mean in the vicinity of *p* = 0, a significantly higher accuracy must be used in order to avoid numerical instabilities.

**Figure 2:**
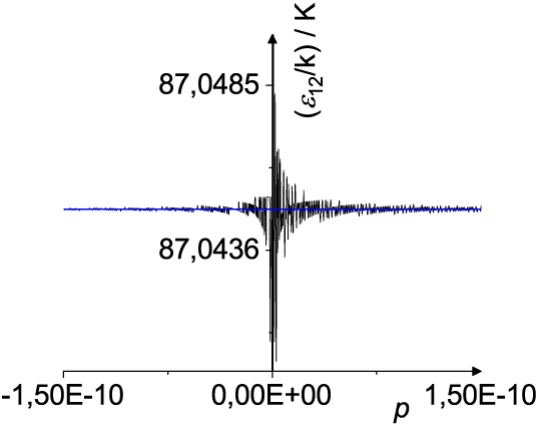
Hölder mean *M_p_* (32.8, 231.0) (black curve), scaled Lehmer mean *L_p_* (32.8, 231.0) and function *U_p_* (32.8, 231.0) (both blue curve) in the vicinity of *p* = 0. The numbers chosen in this example correspond to the potential well depths of neon and xenon. In the case of the Hölder mean the calculations are numerically not stable. According to eq 8 a monotonically increasing function is expected. In contrast the scaled Lehmer mean *L_p_* and *U_p_* behave smooth.

**Table 2:** Potential Energy Parameters of the Noble Gases Used in This Work

Parameter	Reference	He	Ne	Ar	Kr	Xe
10^10^σ/m	Poling et al.^36^	2.551	2.820	3.542	3.655	4.047
	Waldman and Hagler^26^	2.610	2.755	3.350	3.571	3.885
	Sheng et al.^35^	2.644	2.749	3.353	3.579	3.901
(*ε*/*k*)/K	Poling et al.^36^	10.22	32.8	93.3	178.9	231.0
	Waldman and Hagler^26^	10.44	42.00	141.5	197.8	274.0
	Sheng et al.^35^	10.99	42.15	142.95	200.87	279.97

## Mixing Rules for Characteristic Potential Energy Parameters

We concentrate on the potential energy parameters *ε*, σ, and *R*_*m*_ which are used as characteristic properties in many force-field and thermophysical properties calculations. For the potential energy *U*(*R*) we have *U*(σ) = 0 whereas *U*(*R*_*m*_) = −*ε* is the minimum energy value. *R* is the center-of-mass distance. First, we would like to point out two different aspects of the use of the mixing rules. (I) The mixing rules are solely used to obtain the potential parameters *ε*_12_ and σ_12_ of the unlike interaction from *ε*_1_ and *ε*_2_ or σ_1_ and σ_2_, respectively, as good as possible. There is no need to consider any potential energy model *U*(*R*) since due to their definition *ε* and σ do not depend on the special form of *U*(*R*). (II) In a first step the mixing rules are used to obtain *ε*_12_ and σ_12_. Subsequently in a second step a potential energy model *U*(*R*, σ_12_, *ε*_12_) is applied in order to calculate different properties *P* as good as possible. These can be thermophysical properties like the viscosity η_mix_ and the second virial coefficient *B*_mix_ of binary mixtures, respectively, but also the behavior of biomolecules in molecular dynamics simulations. It is important to note that in case (II) two approximations are used and it is difficult to attribute observed deviations between measurement and calculation to only one of the approximations. This has been discussed in full depth by Kříž et al.^8^ Generally in case (II) it would be desirable to have an easily adaptable mixing rule at hand.

There is a large number of mixing rules given in the literature. Some of them are displayed in Table 3. More rules can be found in refs. 14–20 It is worth mentioning that some of these rules require not only *ε* and σ as input parameters for the calculation of *ε*_12_ and σ_12_ but also other properties such as e.g., polarizabilities and magnetic susceptibilities. A very compact representation of a large variety of these mixing rules is given by Diaz Peña et al.^21^ Note that only powers of rational numbers *p* occur in the traditional mixing rules, even though Lennard-Jones and Cook^22^ might have considered the possibility of allowing nonrational numbers as exponents. In Table 3 the mixing rules are also expressed in terms of the Hölder mean *M*_*p*_ and scaled Lehmer mean *L*_*p*_, if appropriate. Note that entries for *M*_*p*_ with *p* = −1, 0, +1 can also be formulated with *L*_*p*_ or *U*_*p*_. Although simple looking this compact representation is new. Inspection of the entries shows that the Hölder mean can be used more often than the Lehmer mean or the function *U*_*p*_. Since the latter one as a mean is restricted to | *p* | ≤ 1 we will not consider *U*_*p*_ in the following. With the help of relations [Disp-formula eq10]–[Disp-formula eq14] the mixing rules written in terms of *M*_*p*_ or *L*_*p*_ can be transformed further, if needed. For instance we can substitute the ratio $M_{0}\left(\sigma_{1},\sigma_{2}\right)/M_{6}\left(\sigma_{1},\sigma_{2}\right)=M_{-6}\left(\sqrt{\sigma_{1}/\sigma_{2}},\sqrt{\sigma_{2}/\sigma_{1}}\right)$ in the Waldman–Hagler mixing rule.

**Table 3:** Frequently Used Mixing Rules (to the Left of the Equal Sign) and Their Equivalent Representation in Terms of the Hölder Mean *M_p_* (*x*_1_, *x*_2_) and Scaled Lehmer Mean *L_p_* (*x*_1_, *x*_2_) (to the Right of the Equal Sign)^a^

Mixing-rule	σ_12_ or *R*_*m*12_	*ε*_12_
Lorentz–Berthelot	(σ_1_ + σ_2_)/2 = *M*_1_(σ_1_,σ_2_)	(*ε*_1_ *ε*_2_)^(1/2)^ = *M*_0_(*ε*_1_, *ε*_2_)
Kříž et al.^8^	${\left((\sigma_{1}^{3}+\sigma_{2}^{3})/2\right)}^{\left(1/3\right)}=M_{3}\left(\sigma_{1},\sigma_{2}\right)$	${\left(2/(\varepsilon_{1}^{-2}+\varepsilon_{2}^{-2})\right)}^{\left(1/2\right)}=M_{-2}\left(\varepsilon_{1},\varepsilon_{2}\right)$
Fender and Halsey^28^	(σ_1_ + σ_2_)/2 = *M*_1_(σ_1_, σ_2_)	2*ε*_1_ *ε*_2_/(*ε*_1_ + *ε*_2_) = *M*_–1_(*ε*_1_, *ε*_2_)
Lee and Sandler^25,40^	${\left((\sigma_{1}^{2}+\sigma_{2}^{2})/2\right)}^{\left(1/2\right)}=M_{2}\left(\sigma_{1},\sigma_{2}\right)$	(*ε*_1_ *ε*_2_)^(1/2)^ = *M*_0_(*ε*_1_, *ε*_2_)
Halgren HHG^27^	$\left(R_{m1}^{3}+R_{m2}^{3}\right)/\left(R_{m1}^{2}+R_{m2}^{2}\right)={\mathrm{L̃}}_{3}\left(R_{m1},R_{m2}\right)=L_{5}\left(R_{m1},R_{m2}\right)$	$4\varepsilon_{1}\varepsilon_{2}/{\left(\varepsilon_{1}^{1/2}+\varepsilon_{2}^{1/2}\right)}^{2}=M_{-1/2}\left(\varepsilon_{1},\varepsilon_{2}\right)$
Waldman–Hagler^26^	${\left((\sigma_{1}^{6}+\sigma_{2}^{6})/2\right)}^{\left(1/6\right)}=M_{6}\left(\sigma_{1},\sigma_{2}\right)$	${\left(\varepsilon_{1}\varepsilon_{2}\right)}^{\left(1/2\right)}\frac{2\sigma_{1}^{3}\sigma_{2}^{3}}{\sigma_{1}^{6}+\sigma_{2}^{6}}=M_{0}\left(\varepsilon_{1},\varepsilon_{2}\right){\left(\frac{M_{0}\left(\sigma_{1},\sigma_{2}\right)}{M_{6}\left(\sigma_{1},\sigma_{2}\right)}\right)}^{6}$
Schnabel et al.^29^ (based on Sikora^16^)	${\left((\sigma_{1}^{12/13}+\sigma_{2}^{12/13})/2\right)}^{\left(13/12\right)}=M_{12/13}\left(\sigma_{1},\sigma_{2}\right)$	$2^{15}\frac{I_{1}I_{2}}{{\left(I_{1}+I_{2}\right)}^{2}}{\left(\varepsilon_{1}\varepsilon_{2}\right)}^{\left(1/2\right)}\times \frac{{\left(\varepsilon_{1}\sigma_{1}^{12}\varepsilon_{2}\sigma_{2}^{12}\right)}^{\left(1/2\right)}}{{\left({\left(\varepsilon_{1}\sigma_{1}^{12}\right)}^{1/13}+{\left(\varepsilon_{2}\sigma_{2}^{12}\right)}^{1/13}\right)}^{13}}=M_{0}\left(\varepsilon_{1},\varepsilon_{2}\right)\frac{M_{0}\left(\varepsilon_{1}\sigma_{1}^{12},\varepsilon_{2}\sigma_{2}^{12}\right)}{M_{1/13}\left(\varepsilon_{1}\sigma_{1}^{12},\varepsilon_{2}\sigma_{2}^{12}\right)}\times \frac{M_{-1}\left(I_{1},I_{2}\right)}{M_{1}\left(I_{1},I_{2}\right)}$

a For *P* = −1, 0, +1, the identities *M_p_* = *L_p_* = *U_p_* are not given explicitly in this table. *I* is the ionization potential.

Since *p* in *M*_*p*_ and *L*_*p*_ is not restricted to rational numbers this new representation is much more flexible. Many mixing rules can be converted into each other just by varying the parameter *p* of the Hölder mean. In Figures 3 and 4 two examples for the binary mixture Ne–Xe are shown. It can be seen, that the results for σ_12_ and *ε*_12_, respectively, can be described by the Hölder mean *M*_*p*_, where the different mixing rules are just a function of the parameter *p*. If we look at σ_12_ *M*_0_(σ_1_, σ_2_) is the mixing rule of Good and Hope,^23^ *M*_12/13_(σ_1_, σ_2_), *M*_1_(σ_1_, σ_2_), *M*_2_(σ_1_, σ_2_), *M*_3_(σ_1_, σ_2_), and *M*_6_(σ_1_, σ_2_) the rules of Sikora,^16^ Lorentz–Berthelot,^24^ Lee and Sandler,^25^ Kříž et al.,^8^ and Waldman and Hagler.^26^ *L*_5_(σ_1_, σ_2_) represents the rule of Halgren.^27^ In the case of *ε*_12_ *M*_–2_(*ε*_1_, *ε*_2_) is the mixing rule of Kříž et al.,^8^ *M*_–1_(*ε*_1_, *ε*_2_) the rule of Fender and Halsey,^28^ *M*_–1/2_(*ε*_1_, *ε*_2_) is given by Halgren,^27^ and *M*_0_(*ε*_1_, *ε*_2_) the Lorentz–Berthelot mixing rule.^24^

**Figure 3:**
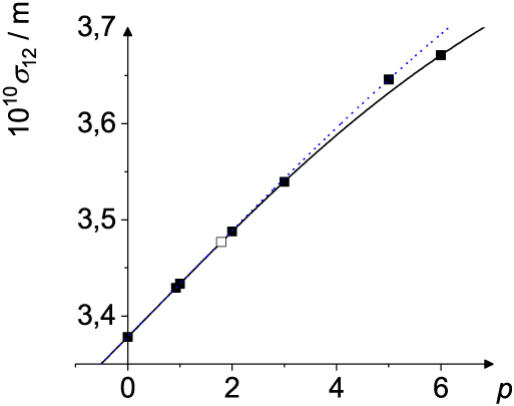
σ_12_ as calculated from the Hölder mean *M_p_* (σ_1_, σ_2_) and scaled Lehmer mean *L_p_* (σ_1_, σ_2_) for σ_1_ = 2.820 × 10^–10^ m (Ne) and σ_2_ = 4.047 × 10^–10^ m (Xe). The potential parameters are taken from Poling et al.^36^ The full line is the Hölder mean as a function of *p*, the blue dotted line the scaled Lehmer mean, the full black squares correspond to the mixing rules of (from left to right) Good and Hope (*M*_0_ = *L*_0_),^23^ Sikora (*M*_12/13_),^16^ Lorentz–Berthelot (*M*_1_ = *L*_1_),^24^ Lee and Sandler (*M*_2_),^25,40^ Kříž et al. (*M*_3_),^8^ Halgren (*L*_5_),^27^ and Waldman and Hagler (*M*_6_).^26^ The open square is obtained from the optimal *p*_σ_ = 1.79 given in Table 4.

**Figure 4:**
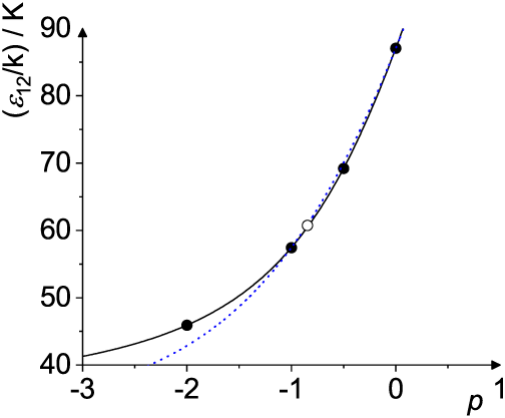
*ε*_12_ as calculated from the Hölder mean *M_p_* (*ε*_1_, *ε*_2_) and scaled Lehmer mean *L_p_* (*ε*_1_, *ε*_2_) as examplified for *ε*_1_/*k* = 32.8 K (Ne) and *ε*_2_/*k* = 231.0 K (Xe). The potential parameters are taken from Poling et al.^36^ The full line is the Hölder mean as a function of *p*, the blue dotted line is the scaled Lehmer mean, the black dots correspond to the mixing rules of (from left to right) Kříž et al. (*M*_–2_),^8^ Fender and Halsey (*M*_–1_ = *L*_–1_),^28^ Halgren (*M*_–1/2_),^27^ and Lorentz–Berthelot (*M*_0_ = *L*_0_).^24^ The open circle is obtained from the optimal *p*_*ε*_ = −0.835 given in Table 4.

This means that by varying *p* one can sweep continuously between the different mixing rules. As mentioned before in the cases *p* → −∞ and *p* → ∞, respectively, it is even possible in a mathematically consistent manner to obtain results for the neat substances.

## Application to Cross Second Virial Coefficients

If we concentrate on the potential parameters only, then the sophisticated rules of Tang and Toennies^18^ work extremely well for obtaining *ε*_12_ and σ_12_. However, they require further input parameters such as the dipole-polarizability α and dispersion interaction energy constants *C*_6_. The simple Lorentz–Berthelot mixing rules fail in this case. However, on the other hand the Lorentz–Berthelot mixing rules are still used very frequently. Surprisingly, in some cases they are hardly worse at describing second virial coeffients, viscosities and mutual diffusion coefficients of binary mixtures when compared to much more complicated mixing rules.^20,29^ As expected, however, considerable deficits are also observed in the description of binary liquid mixtures and soft matter simulations when using the Lorentz–Berthelot mixing rules.^5,30^

In order to show that a continuous variation of the mixing rule might be superior compared to a discrete choice we consider cross second virial coefficients *B*_12_(*T*) of binary noble gas mixtures. To this end a Lennard-Jones (12–6) potential energy function is used.

27 $$U_{12}\left(R\right)=4\varepsilon_{12}\left[{\left(\frac{\sigma_{12}}{R}\right)}^{12}-{\left(\frac{\sigma_{12}}{R}\right)}^{6}\right]$$

Despite the aforementioned deficiencies we choose this function because it is still often used in thermodynamic^29,31^ and force field calculations.^32^ To show how continuous averaging works, we use the Hölder mean *M*_*p*_ as an example. Mainly because many of the traditional mixing rules can be written in form of the Hölder mean, see Table 3. The function *U*_*p*_ is not considered here, as it does not provide mean values for |*p*| > 1. Therefore, the interaction parameters are expressed as the Hölder mean $\varepsilon_{12}=M_{p_{\varepsilon }}\left(\varepsilon_{1},\varepsilon_{2}\right)$ and $\sigma_{12}=M_{p_{\sigma }}\left(\sigma_{1},\sigma_{2}\right)$ . In all cases the optimal parameter *p* is determined by minimizing the squared deviation between the calculated values *B*_12_(*T*) and the smoothed values given by Dymond et al.^33^ To this end, we use the software Wolfram Mathematica,^34^ which employs various mathematical strategies to determine the optimal parameters.

## Results and Discussion

The optimal *p*_*ε*_ and *p*_σ_ as well as the resulting *ε*_12_ and σ_12_ are presented in Table 4 and the resulting *B*_12_(*T*)-curves are shown in Figures 5–14. For comparison high-level *ε*_12_ and σ_12_ given by Sheng et al.^35^ are also displayed in Table 4. It is important to note that the latter values are only intended to reflect the characteristic values of the interaction potential. Therefore, it is not expected that our fitted values do agree with the numbers given by Sheng et al.^35^ Our best fit is always obtained with a nonrational number *p*. The optimal *p*_*ε*_ and *p*_σ_ do, however, depend on the specific binary mixture. *p*_*ε*_ varies between −10.341 (Kr–Xe) and 1.582 (Ar–Kr) whereas *p*_σ_ lies between −7.732 (He–Ne) and 14.222 (Kr–Xe). The cases with *p*_*ε*_ > 0 and *p*_σ_ < 0 have not been considered before in the literature. We did not observe any compliance with the conventional mixing rules listed in Table 3. However, in the cases He–Kr, He–Xe and Ar–Xe *p*_σ_ is roughly 6 which corresponds to the Waldman-Hagler mixing rule. In general, we did not expect agreement with existing mixing rules, because our best *p*-values rely on the specific potential parameters reported by Poling et al.^36^ and the use of the Lennard-Jones potential for calculating the cross second virial coefficients. In order to compare our findings to results obtained from traditional mixing rules we again take the potential parameters given by Poling et al.^36^ and the Lennard-Jones potential and apply the Lorentz–Berthelot and Waldman–Hagler mixing rules, respectively. Obviously in these two cases the agreement between measurement and calculation generally gets worse, see also Figures 5–14. The same trend is observed even in the case that both, the mixing rule and the potential parameters are taken from the special treatment of the rare-gas dimers presented by Waldman and Hagler.^26^ In general none of the tested conventional mixing rules in combination with the Lennard-Jones potential and the potential parameters given by Poling et al.^36^ can reproduce the experimental results with the same accuracy as obtained with the optimized Hölder mean presented in this work.

**Figure 5:**
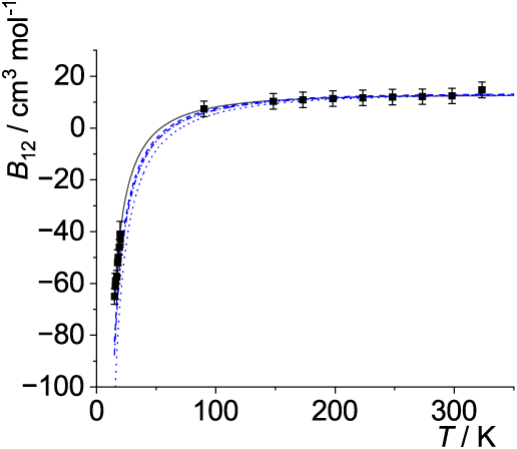
Comparison of cross second virial coefficients *B*_12_(*T*) of He–Ne. Squares: measurements as cited by Dymond et al.,^33^ solid line: calculations with optimal σ_12_ and *ε*_12_ (see Table 4), dash-dotted line: with σ_12_ and *ε*_12_ as obtained from the Lorentz–Berthelot mixing rules, dashed line: with σ_12_ and *ε*_12_ obtained from the Waldman-Hagler mixing rules. The former three are based on the potential parameters given by Poling et al.,^36^ see Table 2. Dotted line: σ_12_ and *ε*_12_ obtained from the Waldman-Hagler mixing rules with potential energy parameters given by Waldman and Hagler,^26^ see Table 2. For all calculations the Lennard-Jones (12–6) potential is used, see eq 27.

**6:**
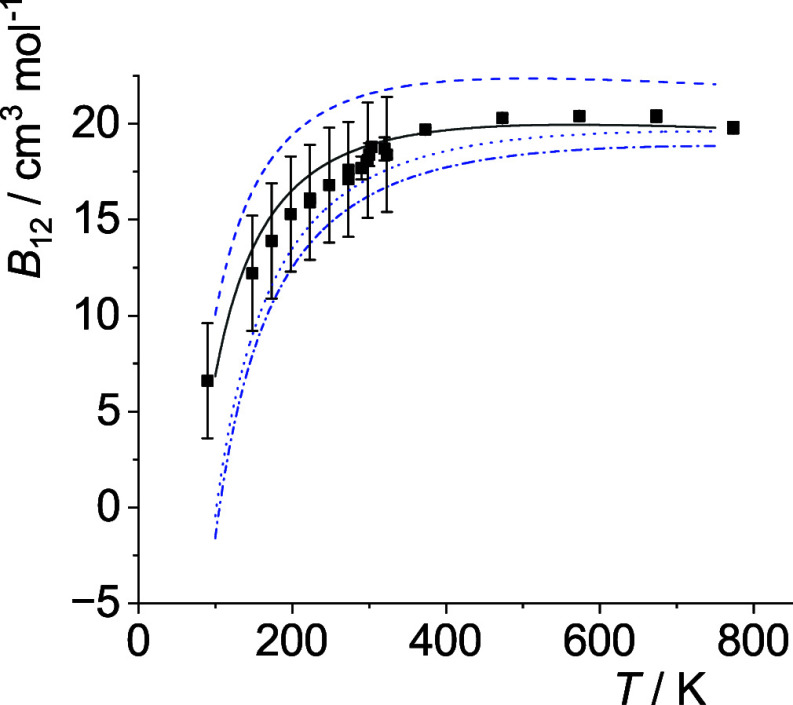
Comparison of cross second virial coefficients *B*_12_(*T*) of He–Ar. The symbols are the same as in Figure 5.

**7:**
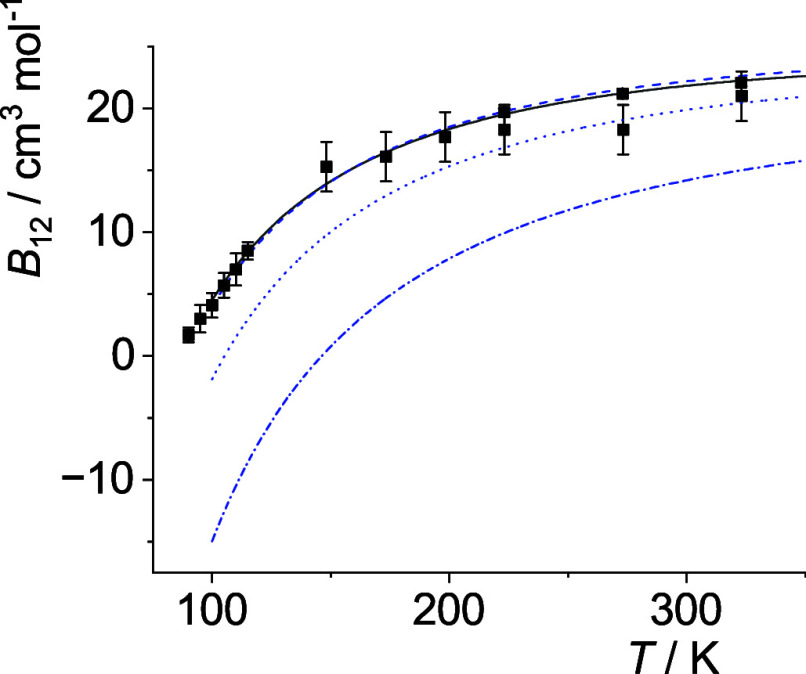
Comparison of cross second virial coefficients *B*_12_(*T*) of He–Kr. The symbols are the same as in Figure 5.

**8:**
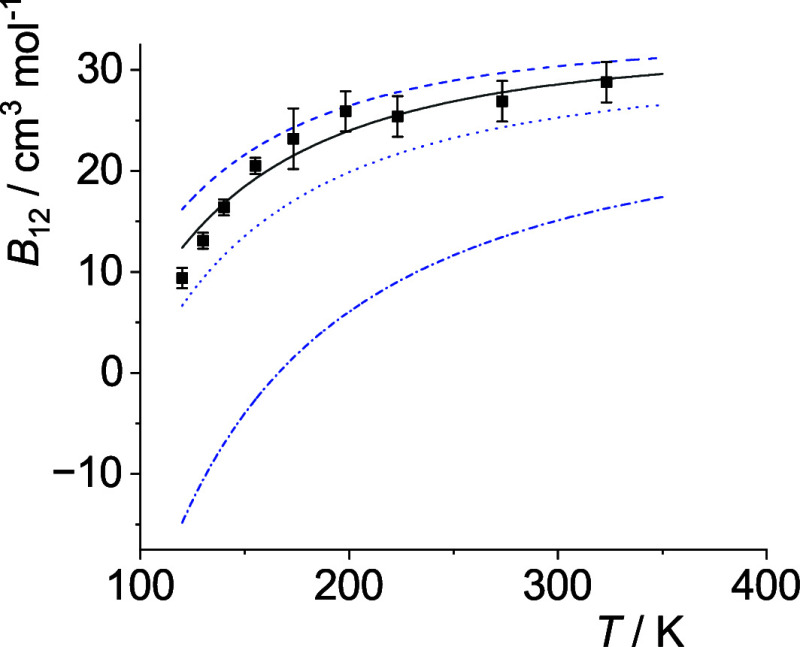
Comparison of cross second virial coefficients *B*_12_(*T*) of He–Xe. The symbols are the same as in Figure 5.

**9:**
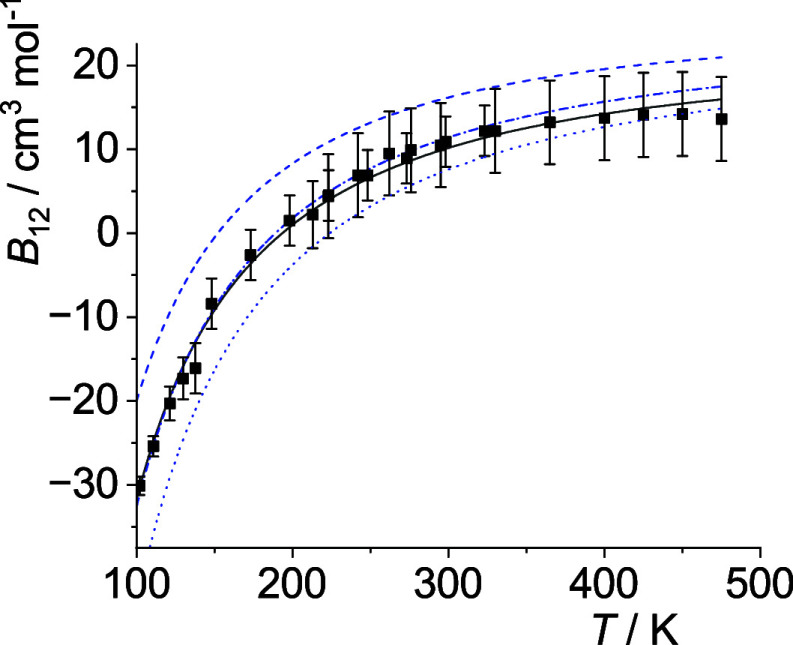
Comparison of cross second virial coefficients *B*_12_(*T*) of Ne–Ar. The symbols are the same as in Figure 5.

**10:**
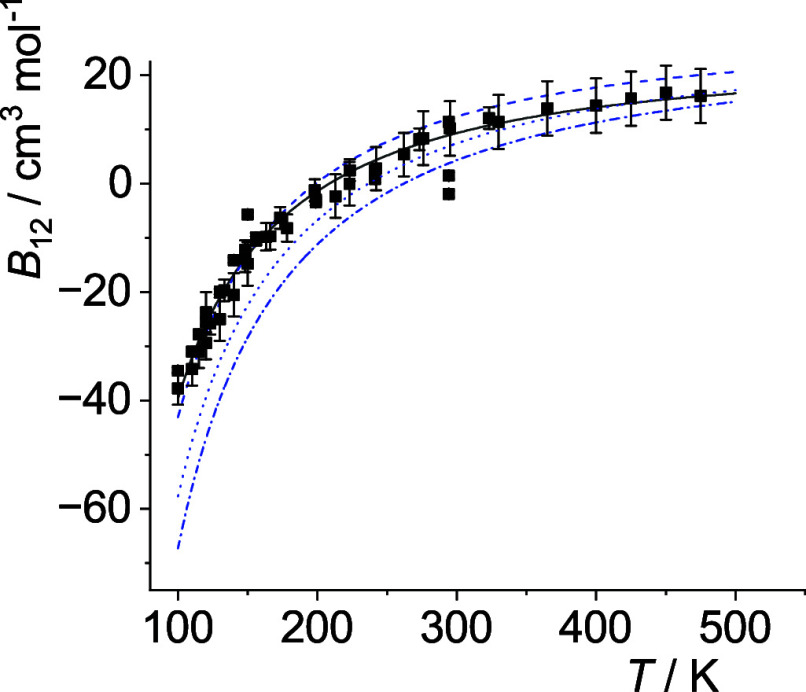
Comparison of cross second virial coefficients *B*_12_(*T*) of Ne–Kr. The symbols are the same as in Figure 5.

**11:**
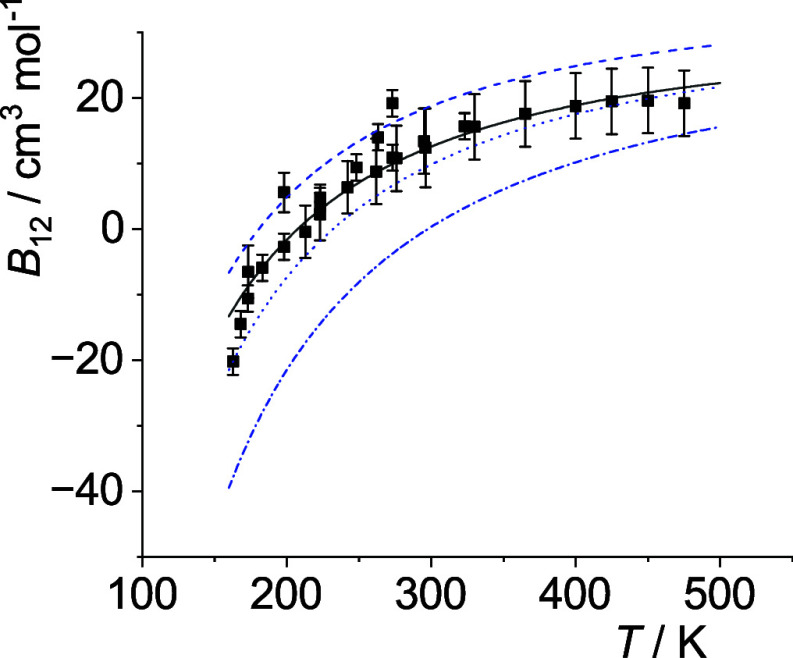
Comparison of cross second virial coefficients *B*_12_(*T*) of Ne–Xe. The symbols are the same as in Figure 5.

**12:**
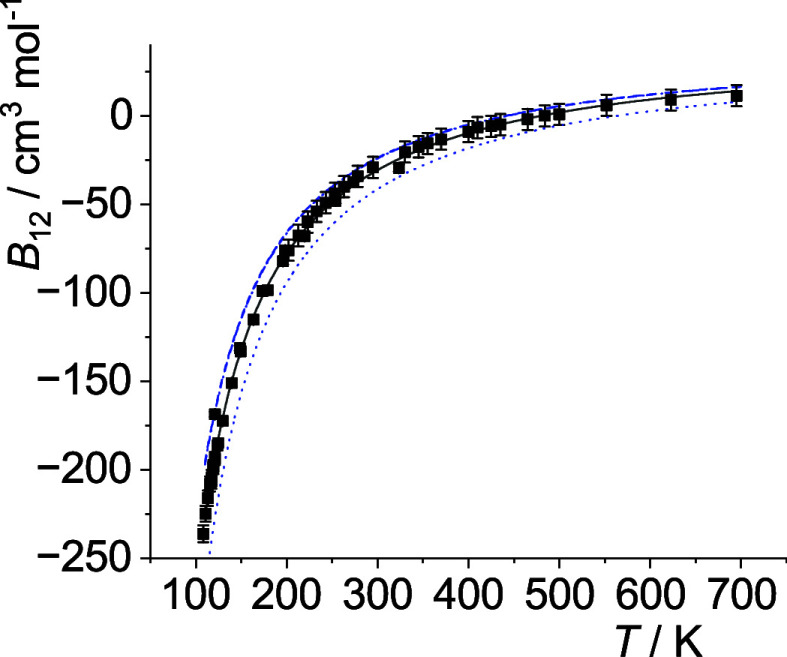
Comparison of cross second virial coefficients *B*_12_(*T*) of Ar–Kr. The symbols are the same as in Figure 5.

**13:**
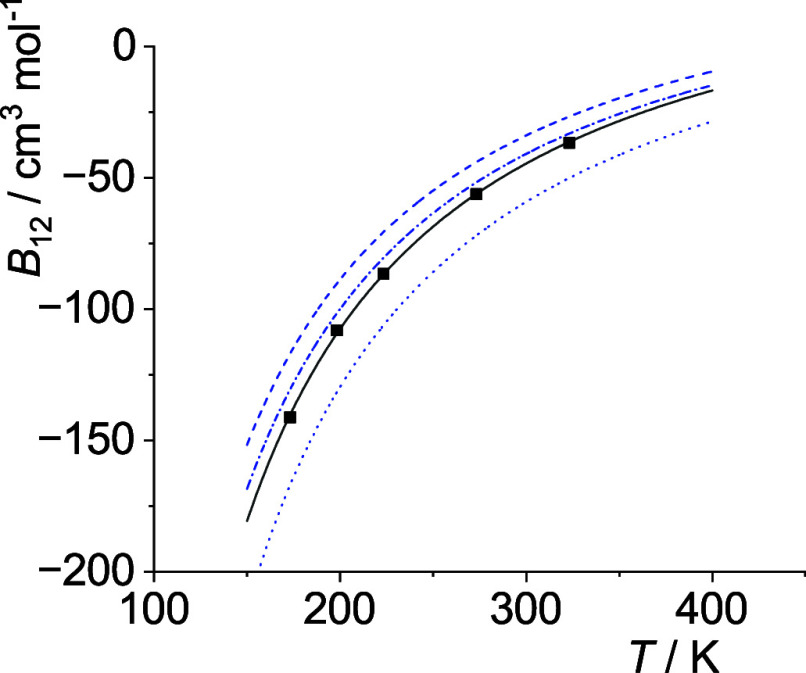
Comparison of cross second virial coefficients *B*_12_(*T*) of Ar–Xe. The symbols are the same as in Figure 5.

**Figure 14:**
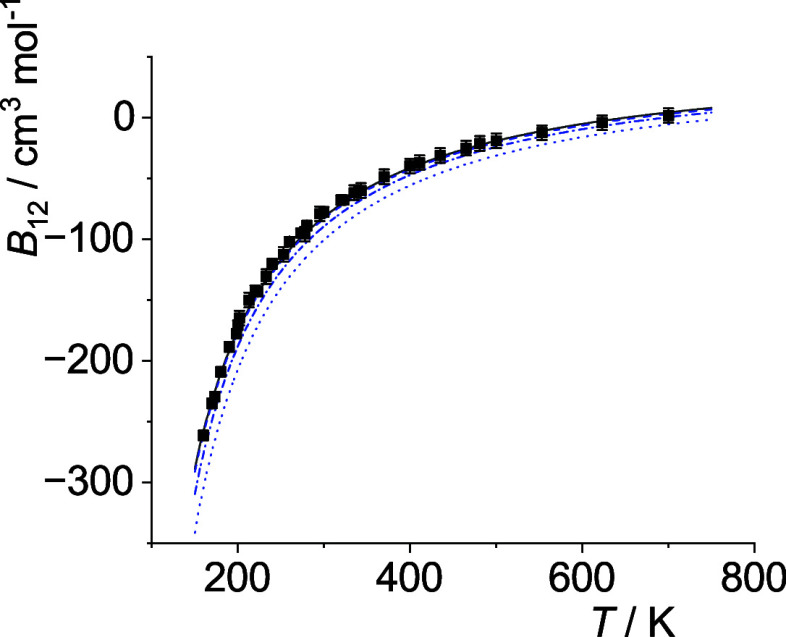
Comparison of cross second virial coefficients *B*_12_(*T*) of Kr–Xe. The symbols are the same as in Figure 5.

**Table 4:** Best *p*-Values *p*_*ε*_ and *p_σ_* and $\varepsilon_{12}=M_{p_{\varepsilon }}\left(\varepsilon_{1},\varepsilon_{2}\right)$ and $\sigma_{12}=M_{p_{\sigma }}\left(\sigma_{1},\sigma_{2}\right)$ (First Row) of the Hölder Mean Obtained by Fitting the Cross Second Virial Coefficient Calculated with the Lennard-Jones (12–6)-potential to the Smoothed Values of *B*_12_(*T*) Given by Dymond et al.^33^ Except for Argon–Xenon 25 Equally Spaced Data Points Are Used^a^

Pair	*T*- range in K	*p*_*ε*_	*p*_σ_	(*ε*_12_/*k*)/K	10^10^σ_12_/m	Standard deviation *s* in cm^3^/mol^–1^
He–Ne	15–350	–0.924	–7.732	15.76	2.657	0.44
				21.03	2.699	
He–Ar	100–750	–0.550	2.464	22.48	3.105	0.85
				29.93	3.110	
He–Kr	100–350	–0.551	5.309	25.57	3.292	0.15
				31.42	3.281	
He–Xe	120–350	–0.598	5.190	25.59	3.601	1.21
				28.09	3.545	
Ne–Ar	100–475	0.168	–3.140	56.60	3.098	1.19
				65.02	3.113	
Ne–Kr	100–500	–0.667	–2.017	60.98	3.157	0.68
				68.52	3.274	
Ne–Xe	160–500	–0.835	1.790	60.72	3.477	2.59
				68.14	3.498	
Ar–Kr	110–700	1.582	–1.427	140.00	3.597	0.92
				161.07	3.496	
Ar–Xe	173.2–323.2	0.174	7.120	149.45	3.844	0.90
				184.41	3.657	
Kr–Xe	150–750	–10.341	14.222	190.04	3.912	2.75
				231.27	3.747	

a In the case of argon–xenon the five data points given in Ref.^33^ are used directly. The potential parameters used for the fit are taken from Poling et al.,^36^ see Table 2. The entries in the second row of *ε*_12_ and *σ*_12_ are reference values given by Sheng et al.^35^

In one aspect the results presented in Table 4 might look disappointing. For each rare gas pair a different *p* of the Hölder mean *M*_*p*_ has to be applied in order to obtain the best fit to the cross second virial coefficients *B*_12_(*T*), meaning that for each pair a different mixing rule works best. But this is somewhat similar to the findings of other researchers^29,37–39^ where mixture dependent parameters ξ_12_ and η_12_ were introduced in the Lorentz–Berthelot mixing rules according to $\varepsilon_{12}=\xi_{12}\sqrt{\varepsilon_{1}\varepsilon_{2}}$ and σ_12_ = η_12_(σ_1_ + σ_2_)/2. Although both ξ_12_ ≈ 1 and η_12_ ≈ 1 strictly speaking the universality of the Lorentz–Berthelot mixing rules immediately gets lost, too. Instead of using any additional factor η_12_ or ξ_12_ we are just adjusting the parameter *p* in order to obtain the best fit. We believe that for practical purposes this procedure is a new and promising tool if mixing rules are used to obtain mixture parameters from data of the pure substances. It can easily be extended and applied for anisotropic systems which are treated via a two-center Lennard-Jones potential or to cases where dispersion interactions are explicitly considered.

## Conclusions

We present the Hölder mean *M*_*p*_(*x*_1_, *x*_2_), the (scaled) Lehmer mean *L*_*p*_(*x*_1_, *x*_2_) and a special function *U*_*p*_(*x*_1_, *x*_2_) which allow for a continuous representation of the mean value *x*_12_ = *f*(*x*_1_, *x*_2_) with *x*_1_, *x*_2_ > 0. Whereas *U*_*p*_ is restricted to | *p* |≤ 1, *M*_*p*_ and *L*_*p*_ can be defined for all real *p*. In the case of *p* = −1, 0, +1 the harmonic, geometric and arithmetic mean is obtained, respectively. Especially the Hölder mean is a new valuable tool in describing mixing rules of potential energy parameters. We show that in this case a flexible mixing rule in terms of the Hölder mean can be used to successfully describe cross second virial coefficients of binary noble gas pairs without any restriction to mixing rules already described in the literature. As the functions *M*_*p*_, *L*_*p*_, and *U*_*p*_ are differentiable, they are particularly easy to use in optimization routines and should be easily included into software packages like GROMACS.
